# RETRACTED: Yosep et al. Cognitive Behavior Therapy by Nurses in Reducing Symptoms of Post-Traumatic Stress Disorder on Children as Victims of Violence: A Scoping Review. *Healthcare* 2023, *11*, 407

**DOI:** 10.3390/healthcare11212858

**Published:** 2023-10-30

**Authors:** Iyus Yosep, Ai Mardhiyah, Gusgus Ghraha Ramdhanie, Citra Windani Mambang Sari, Hendrawati Hendrawati, Rohman Hikmat

**Affiliations:** 1 Department of Mental Health, Faculty of Nursing, Universitas Padjadjaran, Bandung 40132, Indonesia; 2 Department of Pediatric Nursing, Faculty of Nursing, Universitas Padjadjaran, Bandung 40132, Indonesia; 3 Department of Community Nursing, Faculty of Nursing, Universitas Padjadjaran, Bandung 40132, Indonesia; 4 Faculty of Nursing, Universitas Padjadjaran, Bandung 40132, Indonesia

The journal *Healthcare* retracts the article titled “Cognitive Behavior Therapy by Nurses in Reducing Symptoms of Post-Traumatic Stress Disorder on Children as Victims of Violence: A Scoping Review” by Yosep et al. [1]. 

Following publication, concerns were brought to the attention of the publisher regarding a scientific flaw in this article.

Adhering to our complaints procedure, an investigation was conducted, revealing that a proportion of the studies included in the analysis were not suitably relevant to the specific topic of the study. Therefore, the overall findings could not be relied upon. The article is, therefore, retracted. 

This retraction was approved by the Editor-in-Chief of *Healthcare*. 

The authors did not provide a comment on whether they agree or disagree to this retraction. 
